# High Perineal and Overall Frequency of *Staphylococcus aureus* in People Who Inject Drugs, Compared to Non-Injectors

**DOI:** 10.1007/s00284-016-1165-y

**Published:** 2016-11-28

**Authors:** Disa Dahlman, Farnaz Jalalvand, Marianne Alanko Blomé, Anders Håkansson, Håkan Janson, Susanne Quick, Anna C. Nilsson

**Affiliations:** 1 Division of Psychiatry, Department of Clinical Sciences Lund, Lund University, Lund, Sweden; 2 School of Medicine, Örebro University, Örebro, Sweden; 3 Infectious Disease Research Unit, Department of Clinical Sciences, Skåne University Hospital, Malmö, Sweden; 4 Clinical Microbiology, Central Hospital, Växjö, Sweden; 5 Clinical Research Unit, Malmö Addiction Centre, Skåne University Hospital, Södra Förstadsg 35, Plan 4, 205 02 Malmö, Sweden

## Abstract

To investigate the prevalence, distribution, and colonization burden of *Staphylococcus aureus* (*S. aureus*) and MRSA in different body sites among people who inject drugs (PWID) and compare it to a control group consisting of non-injectors. In this cross-sectional survey, 49 active PWID from the needle exchange program (NEP) in Malmö, Sweden, and 60 non-injecting controls from an emergency psychiatric inpatient ward at Malmö Addiction Centre were tested for *S. aureus* (including MRSA) by culture, PCR, and MALDI-TOF. Samples were taken from anterior nares, throat, perineum, and skin lesions if present. Sixty-seven percent of the PWID were colonized with *S. aureus*, compared to 50% of the controls (*P* = 0.08). Perineal carriage was significantly more frequent among PWID than in the control group [37 vs 17%, OR 2.96 (95% CI 1.13–7.75), *P* = 0.03], also after adjusting for sex and age in multivariate analysis [OR 4.01 (95% CI 1.34–12.03)]. Only one individual in the whole cohort (NEP participant) tested positive for MRSA. PWID may be more frequently colonized with *S. aureus* in the perineum than non-injection drug users, and there was a trend indicating more frequent overall *S. aureus* colonization in PWID, as well as higher perineal colonization burden. No indication of a high MRSA prevalence among PWID in Sweden was noted. However, further MRSA prevalence studies among PWID are needed. Knowledge about *S. aureus* colonization is important for the prevention of *S. aureus* infections with high morbidity in PWID.

## Introduction

People who inject drugs (PWID) are at increased risk for superficial and invasive infections with *Staphylococcus aureus* (*S. aureus*), resulting in increased length of hospitalization, mortality and economic cost [16, 45]. In microbiological studies, PWID are more frequently colonized with *S. aureus* than the average population (35% of recent injectors compared to 11% in healthy controls, *P* < 0.05) [42], with nasal colonization in 28–45% [1, 17, 22]. Another concern regarding *S. aureus* is the development and spread of methicillin-resistant *S. aureus* (MRSA) [4]. While Sweden is still a low prevalence country with MRSA prevalence of approximately 1% of *S. aureus* isolates from blood cultures [41], MRSA is a major issue globally [5]. Outbreaks of MRSA among PWID have been reported [14] with carriage rates of 16–20% in previous studies [1, 17, 22].

Association between *S. aureus* nasal colonization and increased risk for infection was reported in the 1930s [44], and supported by several more recent studies [4, 12, 20, 44, 45]. It is suggested that *S. aureus* infections originate from endogenous nasal bacteria that are spread to the skin, which in turn cause infection when the integrity of the skin is compromised [20]. von Eiff et al. [12] showed that in approximately 80% of *S. aureus* bacteremia, the pathogen was identical to the *S. aureus* stem colonizing the subject’s nose.

*S. aureus* colonization (including MRSA) in extra-nasal sites is less investigated. The most frequent sites of colonization are considered to be the skin, the perineum and the pharynx [44]. Several authors stress the need for more studies, and suggest multi-body site sampling [9, 21]. Perineal colonization with *S. aureus* was first described in the 1950s [34], but recent studies are sparse and the clinical importance of perineal carriage in relation to nasal carriage is still unclear.

In longitudinal studies, *S. aureus* carriage patterns have been classified as persistent, intermittent or non-existent [43, 47], with approximately 30% of humans being intermittent nasal carriers [44]. However, figures are varying, and nasal carriage was found in 44% in the general population of Malmö, Sweden, in 2011 [26]. In post-surgical and hemodialysis patients, persistent nasal carriage of *S. aureus* or MRSA has been shown to be a risk factor of *S. aureus* infections [19, 28, 46].

Abundant bacterial growth, i.e. high *S. aureus* colonization burden, has been associated with persistent nasal carriage [39]. In a study by Mermel et al. [24], abundant levels of nasal MRSA was associated with increased likelihood of extra-nasal colonization. Studies on bacterial burden are very sparse, and show incoherent findings regarding risk for infection [19, 38].

The primary aim of this study has been to investigate the prevalence of *S. aureus* among PWID at a needle exchange clinic, and to compare it to a control group consisting of non-injectors. The second aim has been to examine the quantitative level and body distribution of *S. aureus* colonization, and to investigate the prevalence of MRSA in PWID.

## Materials and Methods

### Setting

The study was conducted at the Malmö Needle Exchange Program (Malmö NEP). Malmö NEP is located in the Malmö metropolitan area in Skåne county, Sweden, which has an estimated population of 350,000 inhabitants. A total of 1130 individuals with injection drug abuse are estimated to live in Skåne county, and approximately 600 of them are active visitors of the Malmö NEP each year [15]. Malmö NEP offers sterile injection equipment in exchange for used equipment, as well as certain health care, and surveillance and treatment of viral infections. The Malmö NEP is a part of the Department of Infectious Diseases at Skåne University Hospital in Malmö.

A control group of non-injecting substance dependent patients were recruited from Malmö Addiction Centre (MAC). MAC consists of three inpatient wards, and several outpatient clinics. The control group was recruited from an inpatient ward with 15 beds, treating patients in need of acute detoxification or psychiatric stabilization. The ward is part of the Department of Psychiatry in Malmö, and the care given is thus not strictly somatic. In terms of hygiene standards, patients use hospital clothes but there are no routines concerning personal hygiene for inpatients. Average duration from admission to discharge is four days.

### Design

Participants in the Malmö NEP were randomly invited to participate in the study between May 2014 and September 2015. A control group consisting of hospitalized patients at MAC were also recruited by random invitation. Written informed consent was obtained from all study participants in both groups. Exclusion criteria were psychiatric illness or substance influence severe enough to prevent the patient from providing informed consent. Participants in the control group were excluded if they had injected drugs or been hospitalized abroad in the past 6 months. No compensation for study participation was offered. The study was approved by the Regional Ethical Review Board in Lund (file number 2014/307).

### Microbiological Methods and Data Collection

Samples were collected from the anterior nares, throat, perineum and from skin lesions using Copan E-swabs (480CE; Copan Italia, Brescia, Italy). Separate swabs were used for the different body sites. Nasal, pharyngeal and skin lesion samples were collected by NEP/MAC personnel. Participants in the control group were allowed to handle the swab themselves, strictly supervised by MAC personnel. Perineal samples were in almost 100% of cases collected by the patients in both groups after thorough instructions. The first swab was inserted 1 cm in each nostril and rotated thrice, the second one was streaked twice on one of the tonsil and the third one was streaked on the perineum three times. If there were any skin lesions, they were cleansed prior to sampling from the edges of the lesion. The swabs was stirred for 30 s in the transport medium in separate tubes and thereafter withdrawn. The tubes were forwarded to the Clinical Microbiology Laboratory in Malmö on a daily basis. After the sampling was performed, the participants were interviewed regarding demographic conditions. For the case group, basic medical information regarding drug use was retrieved from the patient chart.

One-hundred µl of each sample was added to a BBL CHROMagar Staph aureus plate (CHROMagar, Paris, France) and 100 µl was added to a Brilliance MRSA 2 agar plate (PO5196A; Oxoid, Basingstoke, United Kingdom). The inoculum was spread on the plates with sterile glass beads and incubated at 35 °C. After 48 h, colonies with typical color appearance (pink on BBL CHROMagar Staph aureus plate and blue on Brilliance MRSA 2 agar plate) were confirmed by a positive agglutination test (Pastorex Staph Plus [Bio-Rad, Hercules, CA, USA]). Suspected MRSA colonies were further verified by *mecA* PCR [32]. In case of growth of colonies with atypical color, Pastorex agglutination and MALDI-TOF were used to rule out atypical *S. aureus* [37]. The number of viable bacteria was measured as colony forming units (CFU). Semi-quantitative evaluation of *S. aureus* growth was made by counting the CFUs, where n CFU > 500 were considered as abundant, 50–499 as intermediate, 1–49 as sparse and 0 as no growth.

The amount of growth was later converted into a hypothetic scale separating abundant growth (=3), from intermediate (=2), sparse (=1) and no growth (=0). To evaluate the total amount of colonization, an accumulative score was calculated for each participant by summing up the scores from each sampling site (nose, throat, perineum and skin lesions) constructing a scale ranging from 0 to 12, with 12 being the most abundantly colonized individuals. The median score in the total amount of colonization was subsequently found to be three, in both the case and control group. Thus, patients with scores ≤3 were considered to be sparsely colonized and scores ≥4 as abundantly colonized.

### Statistical Analysis

Only one variable, overall colonization burden (i.e. *S. aureus* present in any body site cultured), was recoded (see above). To calculate statistical significance, *χ* ^2^ test for dichotomous and Mann–Whitney test for continuous variables were conducted. Unadjusted logistic regression was used for bivariate analysis of categorical variables with more than two options. Variables with *P* < 0.1 in bivariate analysis were further assessed through multivariable logistic regression analysis, adjusting for age and sex. *P* values < 0.05 were considered statistically significant. IBM SPSS statistics, version 22 [18], was used for all the statistical analyses.

## Results

### Population Characteristics

Forty-nine patients from Malmö NEP (69% male, median age 48 years) and 60 control patients from MAC (68% male, median age 52 years) were enrolled in the study (Table 1). Illicit drug use was reported by the case group. Amphetamine and heroin were reported as main drug by 61 and 22%, respectively. Median duration of injection drug use was 22 years (range 1–45 years). Housing status was reported by the case group, and 16% reported unstable housing. Skin lesions were significantly more common in the case group compared to the control group (18 vs 3%, *P* = 0.03).

**Table 1:** Population characteristics

Characteristic	PWID at Malmö NEP *N* (%) Total *N* = 49	Controls at MAC *N* (%) Total *N* = 60	P value
Biological sex			
Male (total *N* = 75, 69%)	34 (69%)	41 (68%)	0.91
Female (total *N* = 34, 31%)	15 (31%)	19 (32%)	
Age in years/median (range)	48 (23–68)	52 (19–77)	0.01*
Subjects with skin lesions (total *N* = 11, 10%)	9 (18%)	2 (3%)	0.03*
Main drug			
Amphetamine	30 (61%)	NA	
Heroin	11 (22%)	NA	
Amphetamine + heroin	2 (4%)	NA	
Other (buprenorphine, methylphenidate, methadone)	6 (12%)	NA	
Duration of injection drug use (year)/median (range)	22 (1–45)	NA	
Unstable housing	8 (16)	NA	

*N* = 49 people who inject drugs (PWID) at Malmö needle exchange program, and *N* = 60 non-injecting substance dependent controls from Malmö Addiction Centre (MAC). Bivariate analysis with *χ* ^2^ test (dichotomous variables)/Mann–Whitney test (continuous variables)

* *P* < 0.05

### Overall *S. aureus* and MRSA Colonization Rates

Colonization in at least one body site, overall *S. aureus* colonization rate, was detected in 67% of PWID, and in 50% of control patients (*P* = 0.08 in bivariate analysis) (Table 2). This difference was not statistically significant in multivariate analysis [OR 1.90 (95% CI 0.82–4.44)] (Table 3). Only one individual (NEP participant) in the total cohort (1%) tested positive for MRSA.

**Table 2:** Bacterial colonization and body distribution with *S. aureus* and MRSA

Characteristic	PWID at Malmö NEP *N* (% of valid answers)	Controls at MAC *N* (% of valid answers)	Sample total *N* (% of total valid answers)	OR (95% CI)	*P* value
*S. aureus* colonization (any body site)					
Yes	29 (67%)	30 (50%)	58 (56%)	2.07 (0.92–4.68)	0.08^+^
No	14 (33%)	30 (50%)	45 (44%)		
*Missing/excluded*	6 excluded	–	6 excluded		
Total valids	43 (100%)	60 (100%)	103 (100%)		
MRSA colonization (any body site)					
Yes	1 (3%)	0	1 (1%)	NA	NA
No	34 (97%)	60 (100%)	94 (99%)		
*Missing/excluded*	14 excluded	–	14 excluded		
Total valids	35 (100%)	60 (100%)	95 (100%)		
*S. aureus* colonization in nares					
Yes	20 (41%)	21 (35%)	41 (38%)	1.28 (0.59–2.79)	0.53
No	29 (59%)	39 (65%)	68 (62%)		
Total valids	49 (100%)	60 (100%)	109 (100%)		
*S. aureus* colonization in throat					
Yes	18 (37%)	20 (33%)	38 (35%)	1.16 (0.53–2.56)	0.71
No	31 (63%)	40 (67%)	71 (65%)		
Total valids	49 (100%)	60 (100%)	109 (100%)		
*S. aureus* colonization in perineum					
Yes	13 (37%)	10 (17%)	23 (24%)	2.96 (1.13–7.75)	0.03*
No	22 (63%)	50 (83%)	72 (76%)		
*Missing/excluded*	14 excluded	–	14 excluded		
Total valids	35 (100%)	60 (100%)	95 (100%)		
*S. aureus* colonization in skin lesions					
Yes	7 (78%)	1 (50%)	8 (73%)	3.50 (0.15–84.69)	0.49^#^
No	2 (22%)	1 (50%)	3 (27%)		
*Missing/excluded*	40 excluded	58 excluded	98 excluded		
Total valids	9 (100%)	2 (100%)	11 (100%)		
Nr of *S. aureus*-colonized locals					
0-Neither nares, throat or perineum	15 (43%)	30 (50%)	45 (47%)		
1-Colonization in one body site	8 (23%)	13 (22%)	21 (22%)	1.23 (0.42–3.61)	0.71
Nares only	1 (3%)	4 (7%)	5 (5%)		
Throat only	4 (11%)	7 (12%)	11 (12%)		
Perineum only	3 (9%)	2 (3%)	5 (5%)		
2-Colonization in two body sites	7 (20%)	13 (22%)	20 (21%)	1.08 (0.36–3.26)	0.90
Nares and throat	2 (6%)	9 (15%)	11 (12%)		
Nares and perineum	4 (11%)	4 (7%)	8 (8%)		
Throat and perineum	1 (3%)	0	1 (1%)		
3-Colonization in nares, throat and perineum	5 (14%)	4 (7%)	9 (9%)	2.50 (0.58–10.70)	0.22
*Missing/excluded*	14 excluded	–	14 excluded		
Total valids	35 (100%)	60 (100%)	95 (100%)		

*N* = 49 people who inject drugs (PWID) at Malmö needle exchange program, and *N* = 60 non-injecting substance dependent controls from Malmö Addiction Centre (MAC). Bivariate analysis with *χ* ^2^ test (dichotomous variables)/unadjusted logistic regression (categorical variables with more than two options)

^+^P < 0.1, * *P* < 0.05

^#^Fisher’s exact test (*N* < 2)

**Table 3:** Variables associated with overall *S. aureus* colonization

Variables	MODEL 1^a^ OR (95% CI)	MODEL 2^b^ AOR (95% CI)
Injection drug use (participation in Malmö NEP vs controls at MAC)	2.07 (0.92–4.68)	1.90 (0.82–4.44)
Age in years		0.99 (0.95–1.03)
Sex (Male vs Female)		0.99 (0.38–2.57)

Unadjusted and adjusted logistic regression analysis. *N* = 103 (excluded *N* = 6)

* *P* < 0.05

^a^Unadjusted

^b^Adjusted for age and sex

### *S. aureus* Distribution in Nasal and Extra-Nasal Sites

*S. aureus* detection rates in specific body sites are presented in Table 2. Fourteen patients in the case group chose to refrain from perineal sampling, and were only included in the nose/throat/skin lesion calculations.

The anterior nares were the most common site of colonization in both groups, 41% in the case group and 35% in the control group, followed by the throat with a 37% detection rate among PWID and 33% in the control group. Perineal colonization was significantly associated with injection drug use in bivariate analysis [37% in PWID vs 17% in controls, OR 2.96 (95% CI 1.13–7.75), *P* = 0.03]. This association remained in multivariate analysis (Table 4), when adjusting for sex and age (OR 4.01 [95% CI 1.34–12.03], *P* = 0.01).

**Table 4:** Variables associated with perineal *S. aureus* colonization

Variables	MODEL 1^a^ OR (95% CI)	MODEL 2^b^ AOR (95% CI)
Injection drug use (participation in Malmö NEP vs controls at MAC)	2.96 (1.13–7.75)*	4.01 (1.34–12.03)*
Age in years		1.04 (0.99–1.09)
Sex (Male vs Female)		2.71 (0.86–8.55)

Unadjusted and adjusted logistic regression analysis. *N* = 95 (excluded *N* = 14)

* *P* < 0.05

^a^Unadjusted

^b^Adjusted for age and sex

Regarding colonization in one body site only, the throat was the most common site in both groups (11 vs 12%). Among the drug injection users it was most common to be colonized in the three carriage sites concurrently, whereas among the non-injectors in two sites (nose and throat). In both groups there were a few individuals with perineal colonization only. The number of colonized locals did, however, not differ significantly between PWID and controls. Several study participants, both at Malmö NEP (8 of 35, 23%) and in the control group (9 of 60, 15%) were colonized in extra-nasal sites only.

### Semi-Quantitative *S. aureus* Colonization Burden

Bacterial burden in different body sites is presented in Table 5, for case and control group respectively. The prevalence of abundant *S. aureus* colonization in the PWID group was found to be 16% (*N* = 8) in the nares, 4% (*N* = 2) in the throat, and 11% (*N* = 4) in the perineum. There was no significant difference between the PWID group and control group in terms of abundant colonization of any specific body site, nor in the over-all colonization burden (as defined in the Methods section). However, there was a trend towards association between abundant perineal colonization and participation in Malmö NEP [OR 4.55 (95% CI 0.77–26.68), *P* = 0.09] in bivariate analysis. Due to a very small number of subjects in the control group (*N* = 2), multivariate analysis was not conducted.

**Table 5:** Semi-quantitative *S. aureus* colonization burden in different body sites

Characteristic	PWID at Malmö NEP *N* (% of valid answers)	Controls at MAC *N* (% of valid answers)	Sample total *N* (% of total valid answers)	OR (95% CI)	*P* value
*S. aureus* colonization burden in nares					
No colonization	29 (59%)	39 (65%)	68 (62%)		
Sparse growth	10 (20%)	5 (8%)	15 (14%)	2.69 (0.83–8.72)	0.10
Intermediate growth	2 (4%)	7 (12%)	9 (9%)	0.38 (0.07–1.99)	0.25
Abundant growth	8 (16%)	9 (15%)	17 (16%)	1.20 (0.41–3.47)	0.74
Total valids	49 (100%)	60 (100%)	109 (100%)		
*S. aureus* colonization burden in throat					
No colonization	31 (63%)	40 (67%)	71 (65%)		
Sparse growth	12 (25%)	17 (28%)	29 (27%)	0.91 (0.38–2.19)	0.83
Intermediate growth	4 (8%)	2 (3%)	6 (6%)	2.58 (0.44–15.02)	0.29
Abundant growth	2 (4%)	1 (2%)	3 (3%)	2.58 (0.22–29.78)	0.45
Total valids	49 (100%)	60 (100%)	109 (100%)		
*S. aureus* colonization burden in perineum					
No colonization	22 (63%)	50 (83%)	72 (76%)		
Sparse growth	7 (20%)	7 (12%)	14 (15%)	2.27 (0.71–7.26)	0.17
Intermediate growth	2 (6%)	1 (2%)	3 (3%)	4.55 (0.39–52.79)	0.23
Abundant growth	4 (11%)	2 (3%)	6 (6%)	4.55 (0.77–26.68)	0.09^*^
*Missing/excluded*	14 excluded	–	14 excluded		
Total valids	35 (100%)	60 (100%)	95 (100%)		
Full-body *S. aureus* colonization burden					
Abundant (median< , i.e. 3<)	8 (23%)	9 (15%)	17 (18%)	1.70 (0.58–4.85)	0.34
Sparse (up to median, i.e. 3 or less)	27 (77%)	51 (85%)	78 (82%)		
*Missing/excluded*	14 excluded	–	14 excluded		
Total valids	35 (100%)	60 (100%)	95 (100%)		

*N* = 49 people who inject drugs (PWID) at Malmö needle exchange program, and *N* = 60 non-injecting substance dependent controls from Malmö Addiction Centre (MAC). Bivariate analysis with *χ* ^2^ test (dichotomous variables)/unadjusted logistic regression analysis (categorical variables with more than two options)

* *P* < 0.1

## Discussion

The present findings with significantly more frequent perineal carriage of *S. aureus* in PWID than in controls are data that to our knowledge are not presented before.

Overall *S. aureus* colonization rate in PWID has been assessed previously. The high percentage of 67% *S. aureus* culture positive PWID at Malmö NEP is, however, not in agreement with the 39% obtained by Bassetti et al., who also found a lower prevalence rate when comparing PWID with non-injectors [6]. These contrasting results may partly be explained by the fact that the maintenance program studied by Bassetti et al. also assisted in injections, in addition to the provision of sterile equipment, while no supervised or assisted injections take place at Malmö NEP. One interpretation of these results could be that it is injections under unclean conditions, rather than the injections themselves, that cause *S. aureus* colonization.

Homelessness is an important factor predisposing for poor hygiene and suboptimal injection conditions. In this study, 16% of PWID reported current unstable housing, which is comparable to two previous studies from Malmö NEP where 40% of PWID reported unstable housing in the past 6 months [10], and 11% of female PWID reported being currently homeless or living in a shelter [33]. In an international perspective, these numbers are low. More than 60% of PWID reported unstable housing in studies from Canada [23] and the U.S. [36]. It is notable that skin lesions were significantly more common among PWID than among controls. This finding is in agreement with previous studies showing that PWID living under poor conditions are at increased risk also for other skin lesions than injections. Common health hazards among homeless people are dermatological problems such as skin infections, eczemas and trauma as well as diseases carried by lice and scabies [3, 31, 40]. It remains unclear whether injections as such increase the *S. aureus* colonization burden among PWID, or if poor living conditions, cramped housing and frequent skin lesions are more important factors, and more research is necessary to further assess main routes of colonization and infection.

*S. aureus* often disseminate from a site of asymptomatic colonization to cause infections, and correlations between commensal carriage and bouts of actual disease have been frequently reported. Intervention programs with nasal decolonization and chlorhexidine body washes decrease *S. aureus* infection rates in surgical and dialysis patients [2, 30]. In the light of PWIDs frequent overall *S. aureus* colonization [1, 17, 22, 42] and high infection rates [16, 45], this group might benefit from similar interventions. Previous studies on chlorhexidine wash as a means of decreasing skin and soft tissue infections (SSTI) in American recruits did not show any significant reduction of the SSTI frequency [13], but was significantly associated with lower frequency of nasal MRSA colonization [25]. These results indicate that PWID, a high-risk population with repeated skin lesions, might benefit from a similar intervention, which to our knowledge has not been conducted. However, a pilot study investigating skin and needle hygiene intervention showed promising results in terms of skin and needle behavioral skills as well as the rate of bacterial infections [29].

Nasal *S. aureus* colonization rate in the current study was 41% in PWID, which is within the range of 28–45% previously reported [1, 17, 22]. The most common site of colonization, independent of carriage in other sites, was the anterior nares. But regarding colonization in one sole body site, the throat was the most common, which also has been noted by other studies from Sweden [27]. Several subjects, both at Malmö NEP and in the control group, were colonized in extra-nasal sites only.

Interestingly, perineal *S. aureus* carriage was statistically more frequent in PWID in comparison to non-injectors (37 vs 17%), which could be a marker for a more extended carriage, and higher colonization burden. Perineal colonization frequency in the case group as well as the control group was higher than in a previous study from 1964 detecting perineal carriage in 13% [7] among 3000 unselected patients admitted to a medical ward. A handful of the individuals (5%) in our cohort were found to be colonized in the perineum only. These results are in agreement with other reports and sole perineal carriage; 3% in a study from 1964 [7] and 8% in one from 1991 [11].

These findings on extra-nasal colonization may be of value when developing clinical routines regarding screening or prophylactic treatment, since screening for MRSA not always include sampling the throat [35] or the perineum. However, it remains to be elucidated whether throat, perineal or multi-local colonization is associated with skin carriage, endogenous infections and transmission to other individuals or not. Future longitudinal studies to evaluate whether *S. aureus* colonization in extra-nasal sites persists over time, and to what extent it’s associated to *S. aureus* infections should be of value.

To our knowledge, this study is the first to present semi-quantification of *S. aureus* in PWID through bacterial cultures. A high level of nasal *S. aureus* colonization in only one swab has pointed at persistent carriage of *S. aureus* which in turn implicates increased risk of infection [28]. Larger studies are however necessary to evaluate possible associations between high colonization burden, immune status, infection risk and multi-site colonization.

Only one PWID in this study was colonized with MRSA, which is considerably lower than the rates of 16–20% reported by previous studies from high endemic areas [1, 17, 22]. Our results are in agreement with the low frequency of MRSA in the general population in Sweden [41] indicating that PWID are not an undetected reservoir of MRSA. However, due to the small sample size, further studies are needed to confirm these primary findings.

This study has limitations that need to be taken into consideration when interpreting the results. First, the sample size is relatively small. Lack of time or interest were often mentioned when a reason was given for refraining from study participation. A quarter of the NEP participants included in the study declined perineal sampling due to lack of time, which may affect the results since the total sample size was small. Furthermore, no compensation for participation was given which also may have affected the choice of partaking. A certain selection of study participants may also occur when recruiting from a NEP, and the results from this study cannot be assumed to concern PWID in general. It has been noted previously that NEP participation might attract PWID with poorer health than the average PWID population [8]. However, we consider the risk of selection bias to be relatively low. The gender distribution, as well as distribution of main drug, among the 49 study participants was similar to that of the entire NEP participant community [oral personal communication, M. Alanko Blomé MD, Medical supervisor of the Malmö NEP, January 2016]. There were no selection criteria when inviting participants to the study, and no information about the study that, to our knowledge, would create bias between study participants and non-participants.

Second, as in all cross-sectional studies, our results may somewhat be affected by the fact that only one single culture was obtained in each individual. The results do show a significantly more frequent perineal *S. aureus* colonization among PWID compared to controls, but this finding is based on sampling at one specific time point and its’ relevance should therefore be viewed with caution. Also, we did not have access to hospital chart data on previous or current clinically significant infections, or antibiotic use. This limitation of patient data do not allow a more thorough analysis of reasons for colonization patterns among PWID and controls, as well as the clinical importance thereof.

Finally, microbiological testing was subject to a limitation since the Clinical Microbiology laboratory performs spa-typing and determines the presence of the PVL-gene on MRSA isolates but not on MSSA. Since the samples resulted only in one MRSA isolate, we were not able to assess PVL-gene and spa-type, something that would have added deeper understanding of possible clonal *S. aureus* spread among PWID (through spa-typing) as well as factors associated with invasive disease (PVL detection).

A strength in the study is the control group consisting of non-injectors with severe substance use disorders in need of hospital care. By using this control group rather than healthy subjects we try to minimize bias in terms of socioeconomic factors, general living conditions and nutritional status. We do not believe that receiving inpatient care was significantly improving the control subjects’ hygiene standards, since average length of hospital treatment is only four days, and the ward does not have specific routines for patients’ personal hygiene. The use of semi-quantitative cultures has, to our knowledge, never have been applied in this group previously and add interesting information.

In conclusion, PWID are significantly more frequently colonized with *S. aureus* in the perineum than non-injectors, and there’s a trend indicating that PWID are also more frequently *S. aureus* colonized in general, and have higher perineal colonization burden. This study does not indicate a high prevalence of MRSA among PWID in southern Sweden, but due to small number of participants further studies are needed to confirm these primary findings. The impact of high frequency of perineal carriage is currently unclear and should be further explored, investigating larger populations with repeated cultures to yield deeper insight in the dynamics of *S. aureus* colonization and its correlation to *S. aureus*-mediated disease.
